# Hyperspectral Vertical Remote Sensing Bridges the Satellite-to-Surface Gap: Unraveling Height-Resolved Evolution Mechanisms of Atmospheric Formaldehyde in China

**DOI:** 10.1021/acs.est.6c02601

**Published:** 2026-05-15

**Authors:** Bowen Chang, Haoran Liu, Muping Chen, Chengxin Zhang, Chengzhi Xing, Qihou Hu, Wei Tan, Qihua Li, Xiangguang Ji, Cheng Liu

**Affiliations:** † State Key Laboratory of Optoelectronic Information Acquisition and Protection Technology, Institute of Physical Science and Information Technology, 12487 Anhui University, Hefei 230601, China; ‡ Department of Precision Machinery and Precision Instrumentation and Key Laboratory of Precision Scientific Instrumentation of Anhui Higher Education Institutes, 12652 University of Science and Technology of China, Hefei 230026, China; § Key Laboratory of Environmental Optics and Technology, Anhui Institute of Optics and Fine Mechanics, Hefei Institutes of Physical Science, 53040 Chinese Academy of Sciences, Hefei 230031, China; ∥ Center for Excellence in Regional Atmospheric Environment, Institute of Urban Environment, Chinese Academy of Sciences, Xiamen 361021, China

**Keywords:** MAX-DOAS, HCHO, generalized additive model (GAM), dominant drivers, vertical evolution, physical mechanisms

## Abstract

Formaldehyde (HCHO) is a hazardous atmospheric pollutant; however, spatiotemporal discrepancies between tropospheric column density (C_HCHO_) and surface concentration (S_HCHO_) hinder the accurate derivation of surface concentrations from satellite observations. Based on 2.5 years of vertical profile measurements from 18 sites within China’s hyperspectral remote sensing network, this study demonstrates that HCHO is predominantly enriched below 0.2 km. Its diurnal variation exhibits a midday-evening bimodal pattern driven by boundary layer dynamics, photochemistry, and anthropogenic emissions. Using Generalized Additive Models (GAMs), we identify the dominant drivers of C_HCHO_ and S_HCHO_ across urban, suburban, and background environments. C_HCHO_ is consistently governed by regional photochemistry (31–52% contribution), whereas S_HCHO_ is primarily controlled by anthropogenic emissions in urban areas (29%) and shifts toward photochemical dominance in suburban (42%) and background (51%) regions. Discrepancies in the response magnitudes of C_HCHO_ and S_HCHO_ to strong emissions and photochemical activity substantially weaken their correlation. By integrating vertical profiles with height-resolved GAMs, we further characterize altitude-dependent mechanisms governing column-to-surface conversion. HCHO below 0.2 km is regulated by local emissions and photochemistry, transitions to photochemical dominance in the middle layer, and becomes increasingly influenced by seasonal variability, large-scale meteorology, and biogenic activity in the upper layer (>1.0 km).

## Graphical abstract

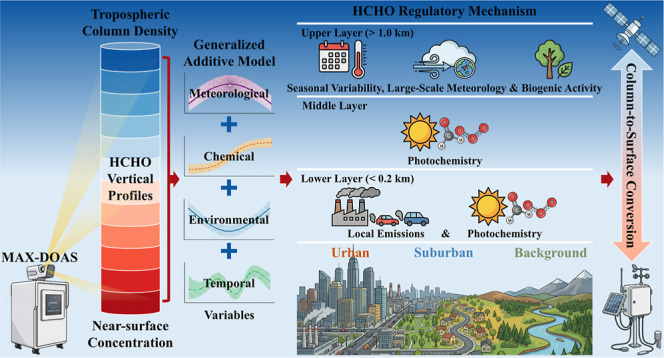

## 1 Introduction

Formaldehyde (HCHO), one of the most abundant carbonyl compounds in the troposphere,^1,2^ serves as a key precursor to ozone (O_3_) formation and plays a central role in atmospheric chemistry.^3–6^ HCHO is also widely recognized as a hazardous air pollutant; epidemiological studies indicate that even short-term exposure to low concentrations can significantly increase the risks of nonaccidental mortality and cardiopulmonary diseases.^7–10^ Ambient HCHO primarily originates from the photochemical oxidation of volatile organic compounds (VOCs),^11–14^ while urban environments are additionally influenced by primary anthropogenic emissions from traffic, combustion, and industrial activities.^15–18^ However, conventional air quality monitoring networks in China lack systematic observations of HCHO,^19^ which limits refined pollution assessments and mechanistic understanding. Consequently, high-spatiotemporal-resolution ground-level HCHO data are urgently needed to support such investigations.

To monitor surface HCHO (S_HCHO_) concentrations, 2,4-dinitrophenylhydrazine (DNPH) derivatization coupled with high-performance liquid chromatography has been implemented at select U.S. Environmental Protection Agency monitoring sites.^20–23^ While this technique offers a low detection limit,^24^ it is susceptible to interference from O_3_ and nitrogen dioxide (NO_2_).^25–27^ Alternative techniques, such as proton-transfer-reaction mass spectrometry (PTR-MS) and differential optical absorption spectroscopy (DOAS),^28,29^ can also quantify HCHO. However, the limited spatial representativeness of these in situ measurements restricts their applicability in regional-scale studies. In contrast, satellite remote sensing (e.g., OMI,^30^ TROPOMI,^31^ GOME-2,^32^ and geostationary instruments such as GEMS and TEMPO)^33,34^ provides long-term, continuous observations of tropospheric HCHO column density (C_HCHO_), offering distinct advantages for global and regional analyses.^35–37^ Nonetheless, C_HCHO_ is not always a reliable proxy for S_HCHO_ due to uneven vertical mixing in the lower troposphere,^19^ which limits the use of satellite data for assessing ground-level pollution. Therefore, elucidating the relationship between C_HCHO_ and S_HCHO_, along with their primary driving factors, is essential for accurately converting column densities into surface concentrations.

The effectiveness of vertical pollutant distributions as indicators of surface air quality exhibits a pronounced spatiotemporal heterogeneity. Although this has been demonstrated by comprehensive field campaigns such as DISCOVER-AQ,^38^ a universal column-to-surface conversion framework remains elusive. Existing studies rely heavily on chemical transport models (CTMs; e.g., GEOS-Chem and TM5)^39,40^ in conjunction with ground-based observations to derive conversion factors, which are subsequently used to estimate surface concentrations from satellite-derived C_HCHO_ for health-risk assessments.^10^ However, simulated vertical profiles are often limited by model resolution and parametrization schemes, leading to an underestimate of near-surface peak concentrations and introducing substantial uncertainties in S_HCHO_ estimates.^41^ Even at high spatial resolution, correlations with ground observations remain moderate (e.g., *r* = 0.59).^40^ Recently, machine-learning approaches, such as neural networks, have been applied to estimate global surface HCHO.^42^ However, their physical interpretability and generalizability remain limited because of the scarcity of in situ vertical profile observations. Therefore, elucidating the vertical evolution of HCHO and its underlying regulatory mechanisms is essential for improving the reliability of the column-to-surface conversion.

Multi-Axis DOAS (MAX-DOAS) enables the retrieval of tropospheric HCHO vertical distributions from scattered sunlight measured at multiple elevation angles,^43–47^ providing critical observational constraints for characterizing the conversion from column densities to surface concentrations.^48^ Leveraging observations from 18 MAX-DOAS sites within China’s ground-based hyperspectral remote sensing network (April 2021–September 2023), this study systematically analyzes the vertical structure and daytime variability of HCHO across diverse regions. By integrating meteorological, temporal, chemical, and environmental variables, Generalized Additive Models (GAMs) are developed separately for C_HCHO_ and S_HCHO_. Comparison of their primary driving factors and distinct nonlinear responses reveals the key mechanisms governing the column-to-surface relationship of HCHO. Furthermore, layered GAMs are employed to elucidate the systematic transitions in HCHO regulatory mechanisms with altitude and their constraining effects on the column-to-surface relationship. This study establishes a physically interpretable framework for C_HCHO_-to-S_HCHO_ conversion from a vertical perspective, providing an observational basis for satellite retrievals and the parametrization of column-to-surface conversion models.

## 2 Materials and Methods

### 2.1 MAX-DOAS HCHO Observations

MAX-DOAS is a passive remote sensing technique based on the Beer–Lambert law for monitoring atmospheric trace gases, aerosols, and other pollutants.^44,45,49^ By analyzing narrowband absorption features in the ultraviolet–visible (UV–vis) spectral range of scattered sunlight measured at multiple elevation angles,^45^ MAX-DOAS retrieves vertical profiles of various atmospheric constituents. This technique has been widely applied owing to its advantages, including rapid response, high sensitivity, versatility, and a high degree of automation.^50^ As summarized in Table 1, HCHO data were retrieved from 18 MAX-DOAS sites between April 2021 and September 2023 within the ground-based hyperspectral remote sensing network led by the University of Science and Technology of China (USTC).^51^ The data set includes HCHO vertical profiles (0–4 km altitude with a vertical resolution of 0.1 km), near-surface HCHO concentrations (S_HCHO_, defined as concentration within 0–0.1 km), and HCHO vertical column densities (C_HCHO_, integrated from 0 to 4 km). The correlation coefficient (r) between C_HCHO_ and S_HCHO_ exhibits substantial intersite variability, generally ranging from 0.44 to 0.84. The monitoring status of each site during the study period is shown in Figure S1, with valid data coverage exceeding 50% (i.e., at least 15 months) for all sites. As illustrated in Figure S2, the study area covers major regions including the Beijing–Tianjin–Hebei (BTH), Yangtze River Delta (YRD), Pearl River Delta (PRD), and Sichuan Basin (SCB). Detailed descriptions of the MAX-DOAS instrumentation and the HCHO vertical profile retrieval algorithm are provided in Text S1.

**Table 1:** Detailed Information on Ground-Based MAX-DOAS Sites

region	city	site	type	longitude (°E)	latitude (°N)	C_HCHO_–S_HCHO_ correlation (r)
BTH	Beijing	UCAS	suburban	116.68	40.41	0.79
		CAMS	urban	116.32	39.95	0.84
	Baoding	WD	suburban	115.15	38.17	0.51
YRD	Huaibei	HNU	urban	116.81	33.98	0.65
	Nanjing	NUIST	suburban	118.72	32.21	0.73
	Hefei	AHU	urban	117.18	31.78	0.69
	Hangzhou	LA	background	119.75	30.30	0.71
PRD	Guangzhou	BLIP01	background	113.79	23.75	0.56
		GIG	urban	113.36	23.15	0.65
		BLIP04	urban	113.26	23.13	0.44
		BLIP05	suburban	113.39	23.05	0.70
		BLIP02	background	113.60	22.75	0.75
	Shenzhen	SZ	suburban	114.00	22.60	0.80
SCB	Chongqing	CQ	urban	106.51	29.60	0.74
other	Huhehaote	IMNU	urban	111.69	40.80	0.79
	Taiyuan	SXU	suburban	112.58	37.63	0.65
	Taian	MT	background	117.11	36.26	0.72
		TA	suburban	117.06	36.20	0.75

### 2.2 Meteorological Data

Meteorological parameters were obtained from the fifth-generation reanalysis data set (ERA5) produced by the European Centre for Medium-Range Weather Forecasts (ECMWF) (available at https://cds.climate.copernicus.eu/datasets, accessed January 1, 2026). Renowned for its high spatiotemporal continuity and physical consistency,^52^ ERA5 has been widely used in studies of atmospheric chemistry and planetary boundary layer (PBL) processes.^53,54^ In this study, both single-level and pressure-level variables at 850 hPa were extracted. The former include boundary layer height (BLH, km), total precipitation (TP, mm), and surface solar radiation downward (SSRD, J m^–2^), whereas the latter include cloud cover fraction (CC), vertical velocity (W, Pa s^–1^), relative humidity (RH, %), temperature (T, K), wind speed (UVS, m s^–1^), and wind direction (UVD, °). The 850 hPa level was selected because meteorological conditions at this geopotential height effectively represent the boundary layer,^55^ providing a robust basis for characterizing C_HCHO_, S_HCHO_, and HCHO vertical profiles. The ERA5 data set has a temporal resolution of 1 h and a spatial resolution of 0.25° × 0.25°. Spatial collocation was performed using the nearest-neighbor method to map each MAX-DOAS site to the corresponding ERA5 grid cell. Temporal alignment between ERA5 data and MAX-DOAS observations was achieved through linear interpolation.

### 2.3 Auxiliary Data

To facilitate HCHO source apportionment, hourly ground-level CO (mg m^–3^) and O_3_ (μg m^–3^) concentrations were obtained from the China National Environmental Monitoring Centre (CNEMC) (available at https://air.cnemc.cn:18007/, accessed January 1, 2026). These data were collected from national monitoring stations located closest to each MAX-DOAS site. To account for biogenic influence on HCHO levels, the MYD13C1 Enhanced Vegetation Index (EVI) product from the Moderate Resolution Imaging Spectroradiometer (MODIS) was used (https://ladsweb.modaps.eosdis.nasa.gov/, accessed January 1, 2026). This data set has a temporal resolution of 16 days and a spatial resolution of 5.5 km. Land cover classification data, used as a key basis for site classification, were obtained from the gridded land cover data set derived from satellite observations and provided by the Copernicus Climate Change Service (C3S LC map; https://cds.climate.copernicus.eu/datasets, accessed January 1, 2026). This data set is updated annually (latest version from 2022) with a spatial resolution of 300 m; data from 2021 and 2022 were used in this study. Demographic data were sourced from the Gridded Population of the World, Version 4 (GPWv4), Revision 11 (https://www.earthdata.nasa.gov/data/catalog, accessed January 1, 2026), which provides quinquennial population estimates from 2000 to 2020 at a spatial resolution of 30 arc-seconds (∼1 km at the equator) based on extrapolation. In this study, population count and density data for 2020 at a resolution of 2.5 arc-minutes were used.

### 2.4 Classification of MAX-DOAS Sites

Given the substantial variability in HCHO sources and pollution characteristics across different environments, the 18 MAX-DOAS sites were systematically classified prior to analyzing their spatiotemporal evolution and underlying mechanisms. Considering that MAX-DOAS measurements have a representative horizontal range of approximately 3–7 km,^56^ the proportions of different land cover types (from the LC map) within a 5 km radius around each site were calculated as the primary classification criterion. This classification was further refined by incorporating population data (GPWv4) and vegetation density (EVI) to ensure a comprehensive characterization of site types. Ultimately, the sites were categorized into three groups: urban, suburban, and background, with detailed results summarized in Table 1.

### 2.5 HCHO Source Apportionment

Atmospheric HCHO originates from both direct anthropogenic and biogenic emissions (i.e., primary sources) and the photochemical oxidation of VOCs (i.e., secondary sources). The mechanisms by which various drivers regulate HCHO concentrations differ substantially depending on the dominant source regime. Because CO exhibits emission patterns that covary with primary HCHO, it is widely used as a tracer of primary combustion sources. In contrast, O_3_ reflects the intensity of atmospheric photochemical activity and covaries with secondary HCHO, thereby serving as an indicator of secondary formation processes.^57–60^ Therefore, prior to statistical modeling, a qualitative assessment of HCHO source characteristics at each site was conducted based on near-surface CO and O_3_ data from CNEMC. This approach facilitates a more robust interpretation of the physical significance and action pathways of influencing factors in subsequent analyses. Details of the HCHO source apportionment method are provided in Text S2.

### 2.6 GAM Development

As a flexible statistical framework, GAMs integrate features of generalized linear models and AMs.^61,62^ By employing link functions, GAMs establish relationships between response variables with various probability distributions and nonparametric smooth functions of explanatory variables. In this study, GAMs were used to systematically evaluate the effects of multiple explanatory variables on the atmospheric HCHO concentrations. Model outputs were interpreted using analysis of variance, providing a concise and flexible characterization of HCHO responses and enabling quantification of the contributions of individual variables (see Text S3 for details).^63^

To maximize the explanatory power and generalizability of the GAMs while ensuring comparability among the C_HCHO_, S_HCHO_, and vertically resolved HCHO models across different site types, an appropriate set of explanatory variables was constructed. Following multicollinearity diagnostics and stepwise selection, the final models included meteorological variables (BLH, TP, SSRD, CC, W, RH, T, UVS, and UVD), a temporal variable (day of study, DOS), chemical tracers (CO and O_3_), and an environmental variable (EVI). To quantitatively assess the influence of each variable, *F* statistics based on changes in deviance was employed. The *F*-value reflects the strength of a given factor in driving HCHO variability, and its proportion relative to the sum of *F*-values across all variables (i.e., relative contribution) was used to identify dominant drivers under different environmental conditions. Details of the F statistic calculation are provided in Text S3.2. Furthermore, partial dependence analysis was applied to characterize the marginal effects of individual variables while accounting for the influence of other covariates. This approach involves fixing the value of a given variable and averaging model predictions over the distribution of all other variables. Given the substantial differences in units and magnitudes among variables, partial dependence results are presented both in absolute terms and as percentages relative to the mean HCHO concentrations. This approach effectively captures the nonlinear response relationships between driving factors and HCHO levels, providing a quantitative basis for subsequent interpretation of the physical mechanism (see Text S3.3 for details).

## 3 Results and Discussion

### 3.1 Vertical Profiles and Temporal Variability of HCHO

Figure 1 presents the vertical profiles and daytime variations of HCHO across urban, suburban, and background sites. Although the daytime BLH remained below 1.3 km for all site types, with HCHO predominantly enriched within the lower PBL, notable differences were observed in vertical gradients and peak altitudes. Urban sites (Figure 1a) exhibited a concentration peak (4.51 ppbv) at approximately 0.2 km, whereas peaks at suburban (Figure 1b) and background (Figure 1c) sites occurred closer to the surface. The mean S_HCHO_ values for urban, suburban, and background sites were 3.69, 3.60, and 3.16 ppbv, respectively, with concentrations at 1.3 km decreasing to less than half of surface levels. Corresponding C_HCHO_ values were 1.14 × 10^16^, 9.52 × 10^15^, and 9.41 × 10^15^ molecules cm^–2^, respectively. Notably, the 0–2 km layer contributed nearly 90% of total C_HCHO_, indicating that the lower troposphere dominates column variability.

**Figure 1:**
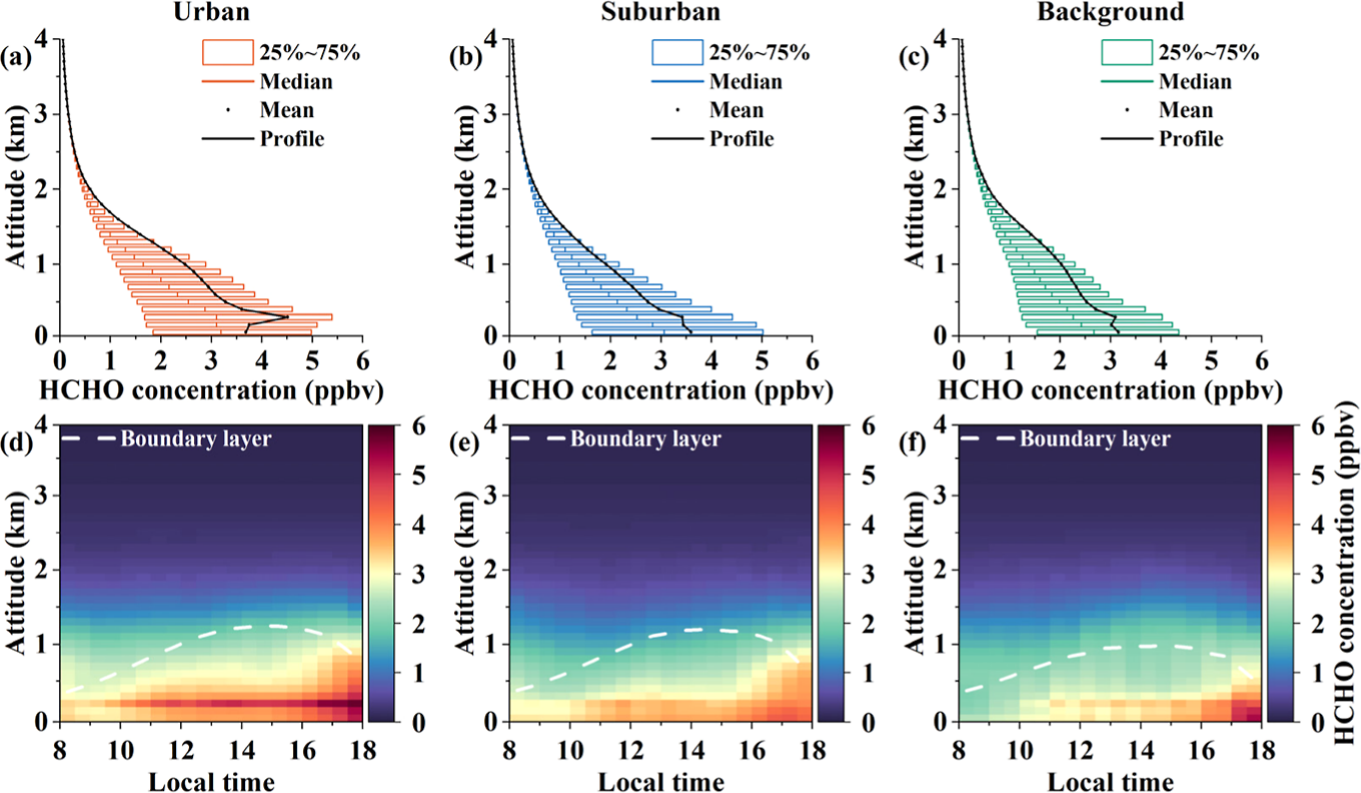
Vertical profiles and daytime variations of HCHO concentrations across different site types. Panels (a,d) represent urban sites, (b,e) suburban sites, and (c,f) background sites.

Figure 1d–f shows that HCHO concentrations increase with the rising BLH during the morning, reaching a midday peak around 12:00–13:00. This pattern reflects the combined effects of boundary layer development, which transports surface-accumulated HCHO upward, and enhanced secondary formation driven by active photochemistry. Although the BLH begins to decline after approximately 15:00, HCHO concentrations continue to increase, reaching a more pronounced peak between 17:00 and 18:00 and forming a distinct bimodal pattern. This behavior suggests that boundary layer compression and downward mixing from the residual layer enhance HCHO accumulation near the surface. This effect is further amplified by evening rush-hour emissions and reduced photochemical loss under weakening solar radiation, resulting in continued HCHO buildup within the boundary layer.

Figure 2 shows that both C_HCHO_ and S_HCHO_ exhibit pronounced temporal variability, with concentrations generally following a seasonal cycle characterized by higher levels in summer and lower levels in winter. This seasonal pattern arises from the combined influences of biogenic volatile organic compound (BVOC) emissions, photochemical production rates, and evolving meteorological conditions.^59,64,65^ In addition, the decoupling between C_HCHO_ and S_HCHO_ becomes more pronounced at higher HCHO concentrations. The proportions of days with weak C_HCHO_–S_HCHO_ correlations in spring (March–May), summer (June–August), autumn (September–November), and winter (December–February) were 55%, 66%, 54%, and 46%, respectively. Across urban, suburban, and background sites, these proportions were 60%, 57%, and 52%, respectively. Together with the substantial spatial variability in these correlations (Table 1), these results indicate that the column-to-surface relationship cannot be adequately represented by uniform scaling or simple end-to-end mapping. Instead, it is primarily governed by vertical distribution structures and the dynamic evolution of HCHO profiles.

**Figure 2:**
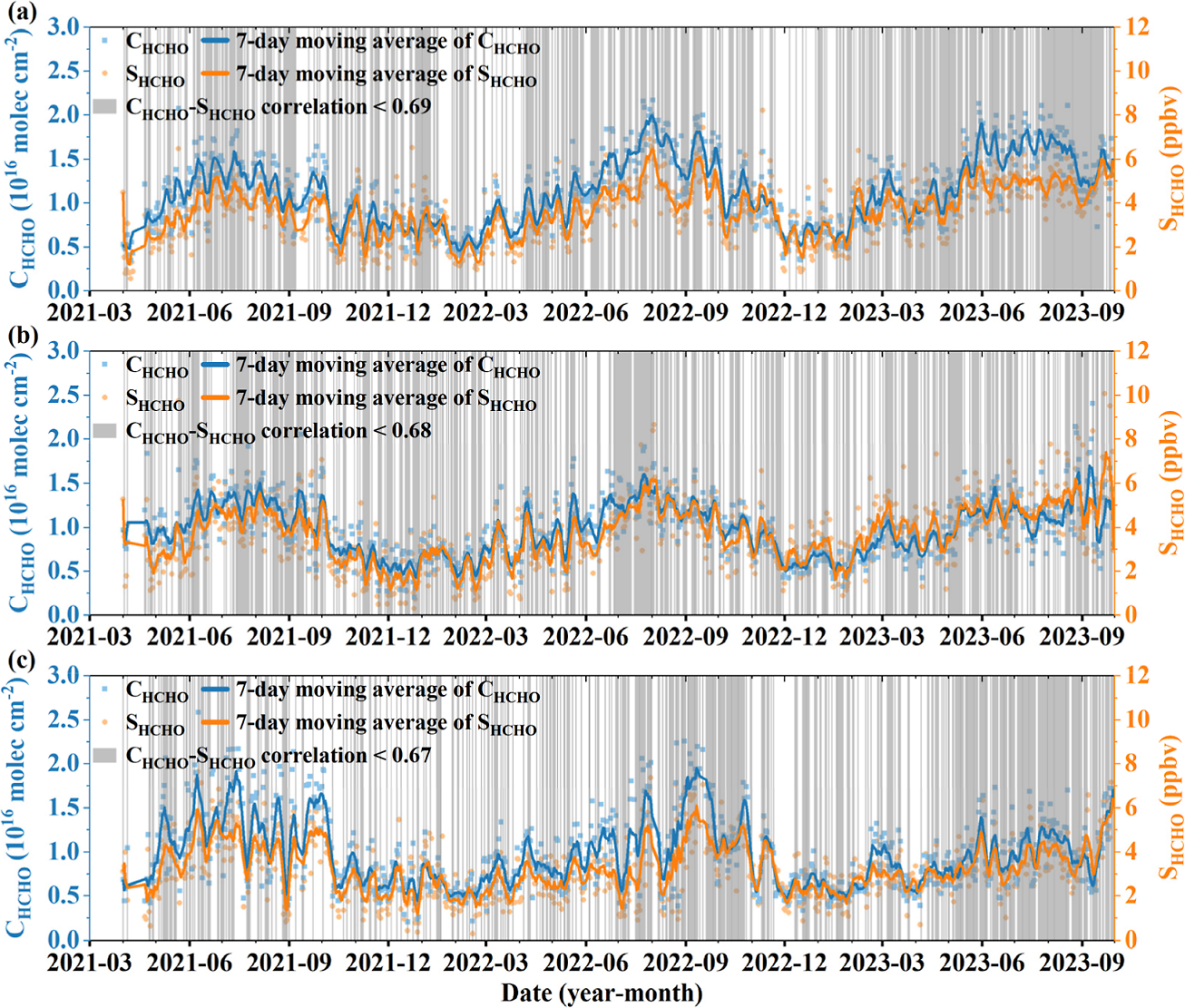
Time series of HCHO concentrations across different site types. Panels (a–c) represent urban, suburban, and background sites, respectively. Blue squares and orange circles denote daily mean C_HCHO_ and S_HCHO_, while solid lines indicate 7 day moving averages. Shaded regions indicate periods when the C_HCHO_–S_HCHO_ correlation falls below the study-period average.

### 3.2 Dominant Factors Influencing the HCHO Column and Surface Concentrations

Figure S4 presents the results of HCHO source apportionment. HCHO concentrations estimated using multiple linear regression (MLR) with near-surface CO and O_3_ show good agreement with MAX-DOAS S_HCHO_ observations (*r* = 0.35–0.78, *p* < 0.01; Figure S5). Urban sites exhibit the highest HCHO concentrations (2.03–5.09 ppbv), whereas the lowest levels occur at background sites (1.58–3.99 ppbv). At urban sites, primary and secondary sources contribute 21–46% and 32–51%, respectively, highlighting the substantial influence of anthropogenic emissions. Most suburban sites show secondary contributions exceeding 45%. At background sites, secondary sources dominate (>53%), while primary contributions remain below 22%, indicating the predominance of regional photochemical processes. Substantial variability in regression intercepts suggests differences in baseline HCHO levels, reflecting the influence of regional background conditions and long-term accumulation. Given the assumptions and limitations of the source apportionment method—particularly its reliance on linear relationships among CO, O_3_, and HCHO—these results should be interpreted as qualitative guidance for subsequent GAM-based mechanistic analysis.

The performance of GAMs for C_HCHO_ and S_HCHO_ across different site types is summarized in Table S2. Correlation coefficients (r) between modeled and observed C_HCHO_ range from 0.74 to 0.80, with explained deviance of approximately 52–64%. This performance exceeds that for S_HCHO_ (*r* = 0.60–0.66, explained deviance <50%). This discrepancy primarily arises because C_HCHO_, as a column-integrated quantity, is more strongly governed by regional meteorology and large-scale processes, resulting in smoother spatiotemporal variability that is more effectively captured by the GAM. In contrast, S_HCHO_ is more sensitive to short-term processes such as local emissions and boundary layer turbulence, leading to greater variability that reduces model performance.

The ranking of drivers based on the GAM F-statistics is shown in Figure 3. O_3_ emerges as the dominant driver for both C_HCHO_ and S_HCHO_ across most site types, with its relative importance increasing from urban to suburban and background environments. A notable exception occurs for S_HCHO_ at urban sites, where CO surpasses O_3_ as the leading factor, highlighting the dominant influence of primary emissions on the near-surface HCHO. In contrast, EVI, T, and DOS consistently act as secondary drivers across all of the site types.

**Figure 3:**
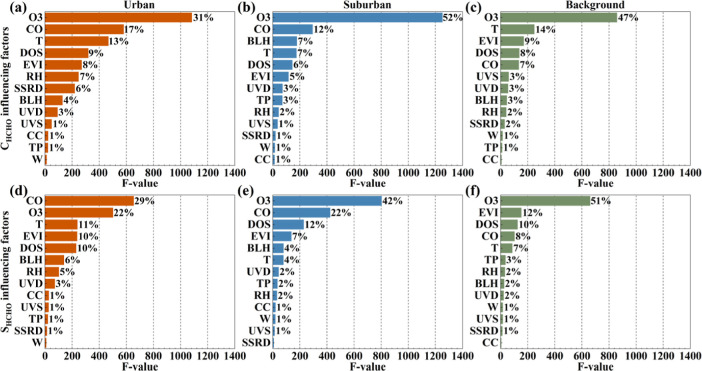
Primary drivers of HCHO concentrations across different site types. Rankings are based on *F*-statistics derived from the GAM, with relative contributions (%) indicated. Panels (a–c) represent C_HCHO_, whereas panels (d–f) represent S_HCHO_. Orange, blue, and green denote urban, suburban, and background sites, respectively.

For C_HCHO_ (Figure 3a–c), O_3_ is the dominant driver at urban sites (relative contribution: 31%), followed by CO (17%) and T (13%), reflecting the combined effects of regional photochemistry and anthropogenic emissions. At suburban sites, C_HCHO_ is strongly influenced by O_3_ (*F* = 1251), with its relative contribution increasing to 52%, alongside consistent contributions from BLH and T, indicating coupled effects of photochemistry and boundary layer dynamics. At background sites, C_HCHO_ is primarily controlled by O_3_ (47%), T (14%), and EVI (9%), underscoring the importance of secondary formation and biogenic processes. In contrast, S_HCHO_ (Figure 3d–f) exhibits stronger environmental heterogeneity. At urban sites, the influence of CO (*F* = 654) exceeds that of O_3_ (*F* = 501), making CO the dominant driver (relative contribution: 29%). At suburban and background sites, S_HCHO_ remains dominated by O_3_ (42 and 51%, respectively), with additional modulation from EVI and DOS. These results indicate that near-surface HCHO is more closely coupled with photochemical and biogenic processes in regions with lower primary emissions.

Overall, C_HCHO_ primarily reflects regional-scale photochemical activity, whereas S_HCHO_ is strongly influenced by primary emissions in urban environments and progressively transitions toward secondary formation and natural processes in suburban and background regions. It should be noted that *F* values represent statistical importance rather than direct physical causality; the underlying mechanisms are further explored through partial dependence analysis in the following section.

Figure 4a–c illustrates the partial dependence of C_HCHO_ on photochemical activity (O_3_), emission intensity (CO), and meteorological and surface factors (T, BLH, and EVI), with other covariates held constant. The regulatory mechanisms exhibit pronounced heterogeneity across site types. While O_3_ remains the dominant driver at all sites, the influences of primary emissions and meteorological conditions are more pronounced at urban and suburban sites. In contrast, background sites are primarily modulated by temperature and biogenic activity. It should be noted that marginal effects at the extremes of the environmental ranges show substantial divergence due to limited data availability. Therefore, the following analysis focuses on data-dense regions of the predictor space.

**Figure 4:**
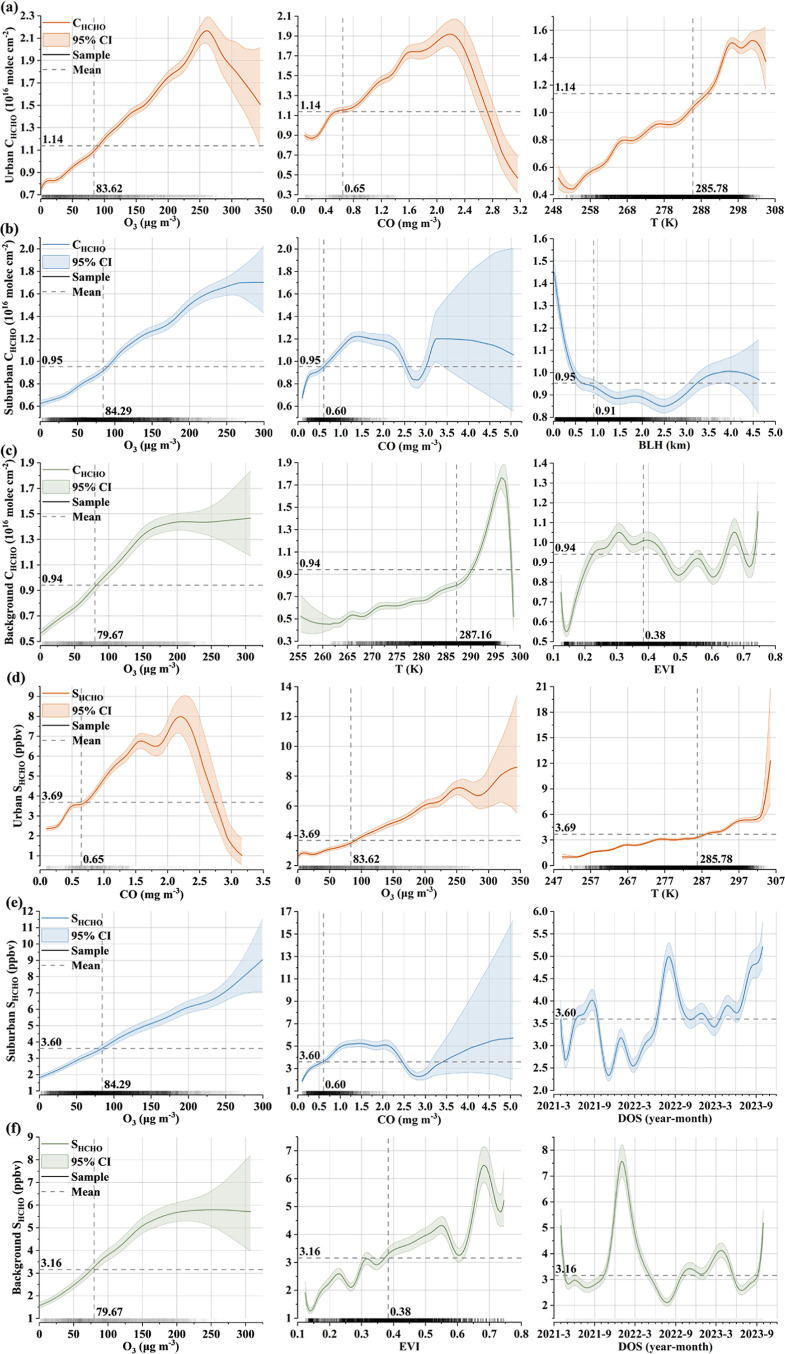
Partial dependence of HCHO concentrations on dominant drivers across different site types. Panels (a–c) represent C_HCHO_, whereas panels (d–f) represent S_HCHO_. Orange, blue, and green denote urban, suburban, and background sites, respectively. Dominant drivers are defined as the top three variables ranked by *F*-statistics in Figure 3. Dashed lines indicate the mean values of each driver and HCHO concentration during the study period.

At urban sites, C_HCHO_ increases monotonically with O_3_, from 7.60 × 10^15^ to 2.17 × 10^16^ molecules cm^–2^, indicating that enhanced atmospheric oxidative capacity promotes the conversion of VOCs to HCHO. CO exerts a nonlinear positive effect, with C_HCHO_ peaking at approximately 1.92 × 10^16^ molecules cm^–2^, reflecting the combined contributions of direct emissions and precursor-related processes. In addition, increasing temperatures (253–296 K) enhances C_HCHO_ by approximately 1.07 × 10^16^ molecules cm^–2^, highlighting the dual roles of increased precursor volatilization and accelerated reaction kinetics. At suburban and background sites, although O_3_ remains the dominant driver, its effect tends to plateau at high concentrations, suggesting saturation of photochemical production efficiency. The positive influence of CO at suburban sites weakens at elevated concentrations, implying potential limitations in precursor availability or oxidation capacity. Meanwhile, the negative dependence on BLH reflects the dilution effect associated with enhanced vertical mixing. At background sites, C_HCHO_ is primarily governed by temperature and vegetation activity, with threshold-like responses of T and EVI underscoring the importance of photochemical production and biogenic emissions.

Figure 4d–f demonstrates that, compared with C_HCHO_, S_HCHO_ exhibits greater sensitivity to local processes. Overall, S_HCHO_ at urban sites is driven by the combined effects of primary emissions, photochemical oxidation, and temperature. In contrast, suburban and background sites are primarily regulated by photochemical activity, biogenic influences, and meteorological conditions. This pattern indicates a systematic shift in S_HCHO_ formation mechanisms from local emission dominance to regional-scale control.

At urban sites, S_HCHO_ shows strong positive responses to both CO and O_3_, reaching peak values of approximately 7.99 and 7.22 ppbv, respectively. This highlights the synergistic interaction between anthropogenic emissions and a highly oxidative environment in increasing near-surface HCHO levels. Concurrently, an increase in temperature consistently enhances S_HCHO_, reaching approximately 5.34 ppbv at 299 K. At suburban sites, S_HCHO_ maintains positive relationships with O_3_ and CO; however, the marginal effect weakens under high-CO conditions, suggesting that photochemical production dominates, with primary emissions acting as a secondary contributor. In addition, the dependence on DOS exhibits a clear seasonal pattern with higher values in summer and lower values in winter. In contrast, background sites emphasize the dominance of the regional processes. Both O_3_ and EVI significantly enhance S_HCHO_, reflecting the contribution of the biogenic emissions. Notably, the response of S_HCHO_ to DOS exhibits an opposite seasonal trend compared with suburban sites, implying that wintertime boundary layer compression and regional transport may play important roles in elevating near-surface HCHO concentrations.

### 3.3 Key Mechanisms Controlling the HCHO Column-to-Surface Relationship

To accurately infer surface concentrations from HCHO column densities, it is essential to elucidate the mechanisms governing the column-to-surface relationship. However, because correlation is an indirect metric, it is not well suited for direct modeling. Instead, this study characterizes variations in the column-to-surface relationship by comparatively analyzing the relative response patterns of C_HCHO_ and S_HCHO_ to individual drivers and their divergences. Among the 13 explanatory variables considered, differences in the relative marginal effects of C_HCHO_ and S_HCHO_ across the three site types are presented in Figures S6–S8. The three variables exhibiting the largest discrepancies are identified as the key drivers influencing the column-to-surface relationship, and their regulatory mechanisms are examined below.

Figure 5a shows that increasing CO concentrations at urban sites enhance both C_HCHO_ and S_HCHO_, but the response of S_HCHO_ is substantially stronger. As CO increases from 0.10 to 1.47 mg m^–3^, the difference in their relative marginal effects (diff) declines sharply from 14.94 pp to −32.98 pp. This pattern indicates that primary emissions disproportionately amplify near-surface HCHO, while the column response remains comparatively muted. Consequently, under high-emission conditions, the dominance of primary sources in S_HCHO_ weakens the column-to-surface consistency, acting as a key driver of the reduced C_HCHO_–S_HCHO_ correlation in urban environments.

**Figure 5:**
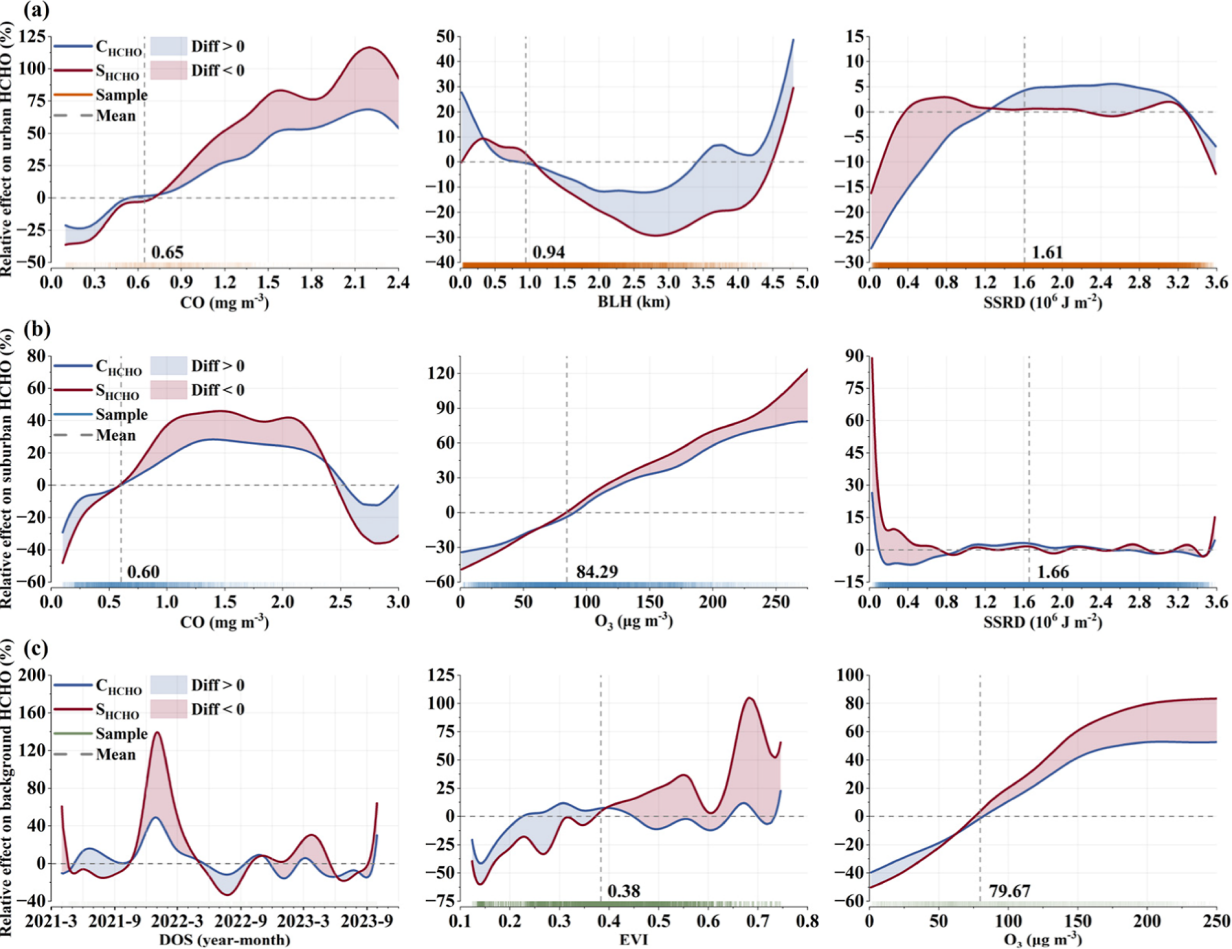
Disparities in the relative marginal effects of dominant drivers on the HCHO column-to-surface relationship across site types. Panels (a–c) represent urban, suburban, and background sites, respectively. Blue and red lines denote the relative marginal effects on C_HCHO_ and S_HCHO_, normalized to their respective mean values. Shaded regions represent the difference (C_HCHO_–S_HCHO_), expressed as Diff (percentage points, pp). Blue and red shading indicate Diff > 0 and Diff < 0, respectively. Vertical dashed lines mark the mean values of each driver.

BLH exhibits a distinct two-stage modulation of the column-to-surface correlation. Under shallow boundary layer conditions (BLH <0.78 km), Diff decreases rapidly from 27.94 pp to −5.52 pp, reflecting strong dilution effects on C_HCHO_ as the boundary layer develops. In contrast, under deeper boundary layer conditions (BLH >0.78 km), Diff increases with BLH, reaching 26.44 pp at 3.66 km. This suggests that enhanced vertical mixing more effectively redistributes and dilutes S_HCHO_, whereas C_HCHO_, as a vertically integrated metric, is less sensitive to further boundary-layer deepening.

Although SSRD is not a primary driver of either C_HCHO_ or S_HCHO_, it exerts a significant influence on their relationship. Under low-radiation conditions (SSRD < 0.34 × 10^6^ J m^–2^), suppressed photochemical production leads to a negative bias in C_HCHO_, driving Diff to −16.10 pp. As SSRD increases, Diff gradually rises and becomes positive (peaking at 6.42 pp). This transition indicates that enhanced radiation-driven photochemistry preferentially strengthens secondary HCHO production throughout the boundary layer, thereby increasing the relative sensitivity of C_HCHO_ compared to S_HCHO_.

Figure 5b shows that the column-to-surface relationship at suburban sites is also governed by the combined effects of primary emissions and photochemical processes, although with an intensity weaker than that at urban sites. Increasing CO leads to a continuous decline in diffusivity, indicating that primary emissions preferentially enhance SHCHO variability under elevated emission conditions. With increasing O_3_, S_HCHO_ exhibits stronger photochemical amplification, causing diff to shift from positive to negative values. This suggests that increases in C_HCHO_ at suburban sites typically require the combined effects of enhanced photochemistry and vertical transport. The influence of SSRD is consistent with that observed at urban sites, where increasing radiation enhances the contribution of secondary formation to C_HCHO_.

Figure 5c indicates that discrepancies in column-to-surface responses at background sites are primarily governed by the coupled effects of regional photochemistry, vegetation activity, and seasonal meteorology. During the summer, Diff remains positive, indicating a stronger response of C_HCHO_ to enhanced secondary formation under high radiation and temperature conditions. In contrast, Diff becomes strongly negative in the winter, reflecting increased sensitivity of S_HCHO_ due to near-surface accumulation and regional transport under weak photochemistry and shallow boundary layer conditions. The influence of the EVI exhibits a segmented response pattern. Under low vegetation cover, C_HCHO_ shows greater sensitivity to increases in EVI, whereas under high EVI conditions, S_HCHO_ responds more strongly, indicating enhanced contributions from BVOC emissions and their near-surface transformations. Overall, the column-to-surface relationship at background sites is predominantly controlled by regional-scale processes rather than by local emissions.

### 3.4 Height-Resolved Regulatory Mechanisms of HCHO and Their Constraints on the Column-to-Surface Relationship

The results presented in Sections 3.2 and 3.3 demonstrate that the dominant drivers of C_HCHO_, S_HCHO_, and their interrelationship differ substantially in both nature and modes of action. To further elucidate these differences, this section develops height-resolved GAMs based on HCHO vertical profiles retrieved from MAX-DOAS observations, aiming to characterize the multivariate regulatory mechanisms governing HCHO vertical distributions and their altitude dependencies. The performance of these altitude-stratified models is shown in Figure S9. By systematically comparing response characteristics across site types and vertical layers, this framework establishes critical mechanistic linkages in the column-to-surface transformation process and provides constraints for the development of physically interpretable conversion methods.

Figure 6 reveals systematic transitions in the regulatory mechanisms of HCHO with altitude across different site types. Overall, HCHO in the near-surface layer (<0.2 km) is jointly influenced by primary emissions (CO) and photochemical production (O_3_). In the middle layer (0.2–1.0 km), the governing regime shifts toward photochemical dominance, with increasing contributions from meteorological factors (T, BLH) and land surface characteristics (EVI). At higher altitudes (>1.0 km), the influence of local emission diminishes, and HCHO is increasingly controlled by seasonal variability (DOS), large-scale meteorological conditions (T), and vegetation activity (EVI).

**Figure 6:**
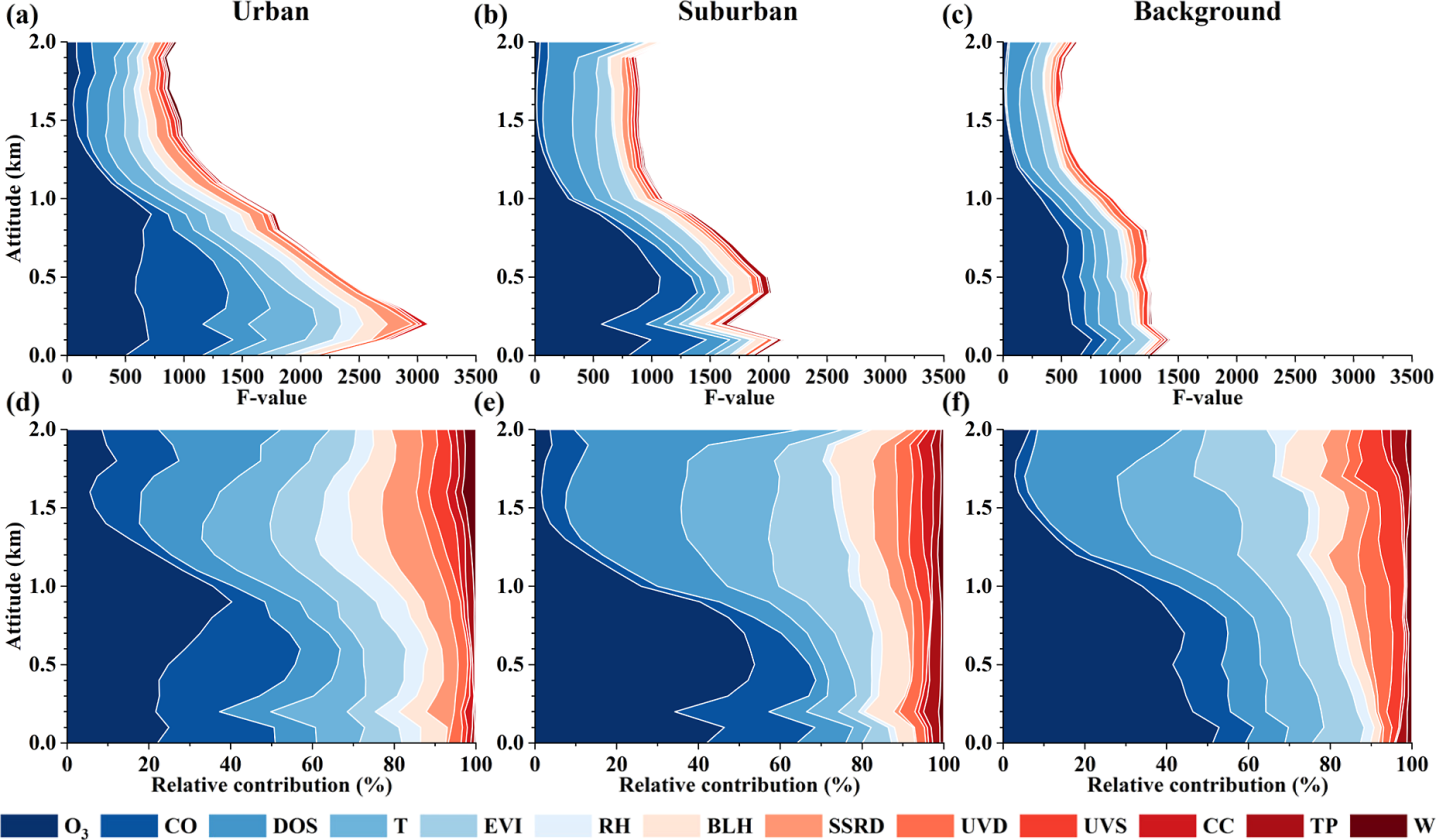
Vertical profiles of the importance (*F*-values) of dominant drivers for HCHO concentrations. The top and bottom rows show absolute *F*-values and their relative contributions, respectively. Columns from left to right represent urban, suburban, and background sites.

At urban sites (Figure 6a,d), near-surface HCHO is sensitive to both CO and O_3_, with relative contributions of 26 and 25% at 0.1 km, respectively, reflecting the combined effects of intensive emissions and active photochemistry. With increasing altitude, the influence of O_3_ remains strong (*F*-values: 583–720), while its relative contribution increases, reaching a maximum of 40% at 0.9 km. This indicates that secondary formation within the boundary layer becomes progressively more important in regulating HCHO. At higher altitudes, HCHO is increasingly dominated by DOS (9–30%) and T (12–17%), suggesting that upper-level HCHO variability is primarily governed by seasonal-scale processes. At suburban sites (Figure 6b,e), HCHO in the lower and middle layers is predominantly driven by O_3_, indicating a stronger influence of regional photochemical production. In the upper layers, DOS becomes the dominant factor, accompanied by notable contributions from T, EVI, and BLH, highlighting the increasing role of natural and meteorological controls. At background sites (Figure 6c,f), the dominance of O_3_ in the lower-to-middle layers is further enhanced, reflecting the importance of regional-scale photochemistry. With increasing altitude, the contributions of meteorological variables (T, BLH) and land surface characteristics (EVI) become more pronounced. In the upper layers, HCHO shows strong dependence on seasonal variability, temperature, and vegetation activity, underscoring the dominant role of natural processes in background environments.

These results indicate that S_HCHO_ primarily reflects near-surface emissions and rapid photochemical processes, whereas C_HCHO_ integrates signals from multiple vertical layers encompassing photochemical, meteorological, and seasonal influences. The altitude-dependent regulatory mechanisms imply that the contribution of different vertical layers to the total column is dynamic rather than fixed, giving rise to the pronounced spatiotemporal heterogeneity observed in the column-to-surface relationship. From a modeling perspective, these findings suggest that future approaches for inferring S_HCHO_ from C_HCHO_ should explicitly account for the vertical heterogeneity. For example, attention-based weighting schemes could be introduced to dynamically adjust the contributions of different drivers according to the site type and altitude. In addition, a layered modeling framework for HCHO could be constructed, followed by vertical integration and consistency evaluation against satellite-derived column observations. Such a hierarchical, physically informed downscaling framework would provide a robust pathway for bridging satellite column measurements and ground-level concentrations, thereby improving the accuracy and interpretability of the surface HCHO estimation.

## Supplementary Material



## Notes

Description of the MAX-DOAS instrumentation and HCHO vertical profile retrieval; description of the HCHO source apportionment method based on MLR; details of the GAM (GAM), including model construction, automatic selection of smoothing parameters, evaluation of variable importance, and partial dependence analysis; variance inflation factor of explanatory variables in the GAM; performance of GAMs for HCHO concentrations across different site types; monitoring status of MAX-DOAS sites; spatial distribution of MAX-DOAS sites; correlation matrix of explanatory variables; HCHO source apportionment results and model performance based on MLR; differences in relative marginal effects of drivers on C_HCHO_ and S_HCHO_ across site types; and performance profiles of height-resolved GAMs across site categories (PDF)
