# Supplementary material for: Hyperspectral Vertical Remote Sensing Bridges the Satellite-to-Surface Gap: Unraveling Height-Resolved Evolution Mechanisms of Atmospheric Formaldehyde in China
Source: Environ Sci Technol. 2026 May 15;60(24):17412–23. doi: 10.1021/acs.est.6c02601 (PMC13296519; doi:10.1021/acs.est.6c02601)
Supplement: Supplementary file 1 [file es6c02601_si_001.pdf]

Supporting Information for the manuscript:

Hyperspectral Vertical Remote Sensing Bridges the Satellite-to-Surface Gap: Unraveling Height-Resolved Evolution Mechanisms of Atmospheric Formaldehyde in China

Bowen Chang[1], Haoran Liu[1,*], Muping Chen[1], Chengxin Zhang[2,*], Chengzhi Xing[3], Qihou Hu[3], Wei Tan[3], Qihua Li[1], Xiangguang Ji[1], Cheng Liu[2,3,4,*]

1. State Key Laboratory of Opto-Electronic Information Acquisition and Protection Technology, Institute of Physical Science and Information Technology, Anhui University, Hefei 230601, China
2. Department of Precision Machinery and Precision Instrumentation, University of Science and Technology of China, Hefei 230026, China
3. Key Laboratory of Environmental Optics and Technology, Anhui Institute of Optics and Fine Mechanics, Hefei Institutes of Physical Science, Chinese Academy of Sciences, Hefei 230031, China
4. Center for Excellence in Regional Atmospheric Environment, Institute of Urban Environment, Chinese Academy of Sciences, Xiamen 361021, China

*Corresponding authors. E-mail: hrl@ahu.edu.cn; zcx2011@ustc.edu.cn; chliu81@ustc.edu.cn

This file contains three parts:

Part I. Supplementary Texts S1-S3 (Pages S2 to S6)
Part II. Supplementary Tables S1-S2 (Page S7)
Part III. Supplementary Figures S1-S9 (Pages S8 to S15)

Part I. Supplementary Texts

Text S1. MAX-DOAS Instrumentation and HCHO Vertical Profile Retrieval

The MAX-DOAS instrument used in this study consists of three core components: a telescope unit, two spectrometers, and a computer system for instrument control and data storage. The two spectrometers cover the ultraviolet (298–409 nm) and visible (420–565 nm) spectral ranges, respectively, with a spectral resolution of approximately 0.45 nm. The telescope collects scattered sunlight at a predefined sequence of elevation angles (1°, 2°, 3°, 4°, 5°, 6°, 8°, 10°, 15°, 30°, and 90°), with a complete scan cycle lasting approximately 15 minutes. Measured spectra are first corrected for offset and dark current to remove instrumental noise.[1,2] Subsequently, spectral analysis is performed using the QDOAS software developed by the Royal Belgian Institute for Space Aeronomy (BIRA-IASB) (https://uv-vis.aeronomie.be/software/QDOAS/, accessed on January 1, 2026).[3] Wavelength calibration is conducted using high-resolution solar reference spectra.[4] QDOAS applies a nonlinear least-squares fitting algorithm to retrieve differential slant column densities (DSCDs),[2] with the zenith spectrum at 90° elevation used as the reference to minimize stratospheric absorption effects. To ensure data quality, spectra with a root mean square (RMS) greater than 2×10^{-3} or a solar zenith angle (SZA) exceeding 75° are excluded. Because DSCDs are influenced by observational geometry and aerosol optical properties, they are converted into vertical column densities (VCDs)—i.e., the integrated HCHO concentration along the vertical path (C_{HCHO})—using air mass factors (AMFs). The VLIDORT radiative transfer model developed by Spurr is employed as the forward model,[5] and HCHO vertical profiles are retrieved from multi-angle DSCDs using the Optimal Estimation Method (OEM).[6-10] The retrieved profiles span altitudes from 0 to 4 km with a vertical resolution of 100 m, with the lowest layer (0–0.1 km) defined as near-surface HCHO concentration (S_{HCHO}). Detailed parameter settings for the retrieval algorithm are provided in Xing et al.[11]

To ensure the robustness and representativeness of the dataset, low-quality profiles with chi-square values (χ^2) greater than 200 or insufficient information content (degrees of freedom < 1) are excluded. Temporal filtering is applied to retain only observations between 08:00 and 18:00 local time. Outliers in C_{HCHO} and S_{HCHO} are identified using the Interquartile Range (IQR) method, and corresponding HCHO profiles outside the range $[Q_1 - 1.5 \times IQR, Q_3 + 1.5 \times IQR]$ are removed. After quality control, 27.96%, 28.67%, and 29.31% of the data are excluded for urban, suburban, and background sites, respectively. A total of 111652, 88199, and 43415 valid profiles are retained for subsequent analysis.

Text S2. HCHO Source Apportionment via Multiple Linear Regression

In this study, a Multiple Linear Regression (MLR) approach was employed to quantitatively partition the various sources of HCHO.[12,13] The mathematical formulation is expressed as:

$$[HCHO] = \beta_0 + \beta_1[CO] + \beta_2[O_3] \quad \text{(S1)}$$

where β_0, β_1, and β_2 represent the regression coefficients, and $[HCHO]$, $[CO]$, and $[O_3]$ denote the observed near-surface concentrations of HCHO, CO, and O_3, respectively. Notably, while previous studies frequently fixed β_0 at 1 ppbv to represent background concentrations, this study accounts for regional variability by flexibly defining β_0 as the 5th percentile of S_{HCHO} during the study period. This value serves as a proximate representation of the regional background level for each site under quiescent emission and photochemical conditions.

Based on this model, the total HCHO concentration is decomposed into the following three

components:

$$P_{primary} = \frac{\beta_1 \overline{CO}}{\beta_0 + \beta_1 \overline{CO} + \beta_2 \overline{O_3}} \times 100\% \quad \text{(S2)}$$

$$P_{secondary} = \frac{\beta_2 \overline{O_3}}{\beta_0 + \beta_1 \overline{CO} + \beta_2 \overline{O_3}} \times 100\% \quad \text{(S3)}$$

$$P_{background} = \frac{\beta_0}{\beta_0 + \beta_1 \overline{CO} + \beta_2 \overline{O_3}} \times 100\% \quad \text{(S4)}$$

where $\overline{CO}$ and $\overline{O_3}$ represent the mean concentrations of CO and O_3. The terms $P_{primary}$, $P_{secondary}$, and $P_{background}$ represent the relative contributions from primary emissions, secondary formation, and background levels to the total HCHO concentration, respectively.

Text S3. Generalized Additive Models (GAMs)

Prior to Generalized Additive Model (GAM) construction, candidate explanatory variables were rigorously screened and validated to prevent severe multicollinearity from compromising model stability and interpretability. First, a pairwise correlation analysis was conducted to preliminary identify potential redundancies among variables via a Pearson correlation (r) matrix. Subsequently, the Variance Inflation Factor (VIF) was employed to quantitatively assess multicollinearity, reflecting the degree to which an explanatory variable can be linearly explained by others. A higher VIF indicates more significant multicollinearity. Detailed results for r and VIF are provided in Figure S3 and Table S1. The analysis confirms that no significant multicollinearity exists among the 13 explanatory variables integrated into the final GAM ($-0.39 \leq r \leq 0.55$, VIF < 1.99), ensuring a sound statistical foundation and physical interpretability for the subsequent importance assessment and partial dependence analysis.

The GAM modeling process consists of three primary stages:

1. Model Configuration: Given that atmospheric HCHO concentrations are positive and exhibit a distinct right-skewed distribution, the GAM was constructed using a Gamma distribution with a log-link function.[14-16] The optimal smoothing parameters for each penalized spline were automatically determined via Generalized Cross-Validation (GCV).

2. Performance and Importance Evaluation: Based on the optimal model, the fitting and predictive performance were evaluated. The F-value of each explanatory variable was calculated to characterize its relative importance.

3. Partial Dependence Analysis: Partial dependence analysis was performed to quantitatively characterize the non-linear response of HCHO concentrations to each explanatory variable.

The mathematical structure of the GAM is expressed as follows:

$$\log(\mu_i) = \beta_0 + \sum_{j=1}^{p} f_j(x_{ij}) \quad \text{(S5)}$$

where μ_i is the expected HCHO concentration for the i-th sample; β_0 represents the model intercept; x_{ij} is the value of the j-th explanatory variable for the i-th sample; $f_j(\cdot)$ denotes the smoothing function (represented by penalized splines) used to capture the non-linear relationship between the variable and HCHO concentration; and p is the total number of explanatory variables. Details regarding the smoothing penalty parameters are provided in Text S3.1.

Model predictive performance was quantitatively assessed using the following metrics: r, measuring linear consistency between predicted and observed values; percentage of deviance explained, representing the overall explanatory power; Root Mean Square Error (RMSE); Mean

Absolute Error (MAE); and Mean Absolute Percentage Error (MAPE). The formulas are as follows:

$$Deviance\ explained = \frac{DEV_{null} - DEV_{full}}{DEV_{null}} \times 100\% \tag{S6}$$

$$RMSE = \sqrt{\frac{1}{n}\sum_{i=1}^{n}(\hat{y}_i - y_i)^2} \tag{S7}$$

$$MAE = \frac{1}{n}\sum_{i=1}^{n}|\hat{y}_i - y_i| \tag{S8}$$

$$MAPE = \frac{100}{n}\sum_{i=1}^{n}\left|\frac{\hat{y}_i - y_i}{y_i}\right| \tag{S9}$$

where n is the number of samples; y_i and $\hat{y}_i$ represent the observed and predicted values, respectively; DEV_{null} denotes the deviance of the null model (containing only the intercept), reflecting the fitting error without explanatory variables; and DEV_{full} denotes the deviance of the final GAM, reflecting the residual error after incorporating all explanatory variables and their respective smoothing terms.

Text S3.1. Automatic Selection of Smoothing Penalty Parameters in the GAM

Each smoothing function in the GAM is associated with a smoothing penalty parameter (λ), which is used to strike a balance between model fitting accuracy and the degree of smoothness. A smaller λ allows for greater flexibility in the functional form, whereas a larger λ increases the smoothing penalty to mitigate overfitting. The optimal smoothing parameters are determined by minimizing the GCV criterion, which aims to control the effective degrees of freedom while maintaining fitting precision. The general form of GCV is expressed as:

$$GCV \approx \frac{\frac{1}{n}\sum_{i=1}^{n}(y_i - \hat{y}_i)^2}{\left(1 - \frac{edf}{n}\right)^2} \tag{S10}$$

where n is the sample size, y_i and $\hat{y}_i$ represent the observed and predicted values, respectively, and edf denotes the effective degrees of freedom of the model.

In the practical implementation, the initial value for each smoothing term's λ was set to 1, and a candidate parameter set was constructed containing 25 logarithmically spaced values ranging from 10^{-2} to 10^{2}. Subsequently, an optimization strategy akin to variable-wise coordinate descent was employed: while holding the λ values of other variables constant, the algorithm traversed all candidate λ values for the current variable to identify the one that minimized the GCV. This process was performed sequentially for all variables and repeated for two full cycles to ensure stable convergence of the parameters.

Text S3.2. Importance Assessment of Explanatory Variables in the GAM

To evaluate the relative importance of individual variables, a full model incorporating all candidate explanatory variables is first fitted to determine its residual deviance (DEV_{full}) and effective degrees of freedom (edf_{full}). Subsequently, for each specific explanatory variable, a reduced model is constructed by omitting that variable, and the corresponding residual deviance (DEV_{red}) and effective degrees of freedom (edf_{red}) are calculated. The F-statistic, which characterizes the significance of the contribution of the omitted variable to the model's explanatory power, is defined as:

$$F = \frac{\left(DEV_{red} - DEV_{full}\right)/\left(edf_{full} - edf_{red}\right)}{DEV_{full}/\left(n - edf_{full}\right)} \tag{S11}$$

Text S3.3. Partial Dependence Analysis of GAM Explanatory Variables

The partial dependence function for each explanatory variable in the GAM can be formulated as:

$$PD(x_j) = E_{X_{-j}}[\mu(x_j, X_{-j})] \quad \text{(S12)}$$

where $PD(x_j)$ represents the average marginal contribution of the j-th explanatory variable to the HCHO concentration when it takes the value x_j; $\mu(\cdot)$ denotes the expected prediction of the GAM given a set of independent variables; and X_{-j} represents the set of all other explanatory variables excluding the j-th variable. This definition captures the statistical relationship between the model response and a specific variable by marginalizing over the distribution of the remaining variables in the dataset.

In the numerical implementation, a computational grid of values is first constructed for each explanatory variable based on its observed distribution in the sample. Subsequently, the values of the target variable are iteratively replaced by these grid values while holding all other variables constant at their original observed values. These modified datasets are then fed into the fitted GAM, and the resulting predictions are averaged across all samples to yield the partial dependence value for each grid point. To characterize uncertainty, the upper and lower bounds of the partial dependence curves are calculated at each grid point based on the confidence intervals derived from the model's prediction variance.

References

1 Hönninger, G., von Friedeburg, C. & Platt, U. Multi axis differential optical absorption spectroscopy (MAX-DOAS). *Atmos. Chem. Phys.* **4**, 231-254 (2004). https://doi.org/10.5194/acp-4-231-2004

2 Stutz, J. & Platt, U. Numerical analysis and estimation of the statistical error of differential optical absorption spectroscopy measurements with least-squares methods. *Appl. Opt.* **35**, 6041-6053 (1996). https://doi.org/10.1364/AO.35.006041

3 Javed, Z. *et al.* Investigating the impact of Glyoxal retrieval from MAX-DOAS observations during haze and non-haze conditions in Beijing. *Journal of Environmental Sciences* **80**, 296-305 (2019). https://doi.org/10.1016/j.jes.2019.01.008

4 Chance, K. & Kurucz, R. L. An improved high-resolution solar reference spectrum for earth's atmosphere measurements in the ultraviolet, visible, and near infrared. *Journal of Quantitative Spectroscopy and Radiative Transfer* **111**, 1289-1295 (2010). https://doi.org/10.1016/j.jqsrt.2010.01.036

5 Spurr, R. J. D. VLIDORT: A linearized pseudo-spherical vector discrete ordinate radiative transfer code for forward model and retrieval studies in multilayer multiple scattering media. *Journal of Quantitative Spectroscopy and Radiative Transfer* **102**, 316-342 (2006). https://doi.org/10.1016/j.jqsrt.2006.05.005

6 Clémer, K. *et al.* Multiple wavelength retrieval of tropospheric aerosol optical properties from MAXDOAS measurements in Beijing. *Atmos. Meas. Tech.* **3**, 863-878 (2010). https://doi.org/10.5194/amt-3-863-2010

7 Hendrick, F. *et al.* Four years of ground-based MAX-DOAS observations of HONO and NO_2 in the Beijing area. *Atmos. Chem. Phys.* **14**, 765-781 (2014). https://doi.org/10.5194/acp-14-765-2014

8 Wang, T. *et al.* Evaluation of tropospheric SO_2 retrieved from MAX-DOAS measurements in Xianghe, China. *Atmos. Chem. Phys.* **14**, 11149-11164 (2014). https://doi.org/10.5194/acp-14-11149-2014

9 Chan, K. L., Wiegner, M., Wenig, M. & Pöhler, D. Observations of tropospheric aerosols and NO_2 in Hong Kong over 5years using ground based MAX-DOAS. *Science of The Total Environment* **619-620**, 1545-1556 (2018). https://doi.org/10.1016/j.scitotenv.2017.10.153

10 Jiao, P. *et al.* A dataset of ground-based vertical profile observations of aerosol, NO_2, and HCHO from the hyperspectral vertical remote sensing network in China (2019–2023). *Earth Syst. Sci. Data* **17**, 3167-3187 (2025). https://doi.org/10.5194/essd-17-3167-2025

11 Xing, C. *et al.* Identifying the wintertime sources of volatile organic compounds (VOCs) from MAX-DOAS measured formaldehyde and glyoxal in Chongqing, southwest China. *Science of The Total Environment* **715**, 136258 (2020). https://doi.org/10.1016/j.scitotenv.2019.136258

12 Friedfeld, S. *et al.* Statistical analysis of primary and secondary atmospheric formaldehyde. *Atmospheric Environment* **36**, 4767-4775 (2002). https://doi.org/10.1016/S1352-2310(02)00558-7

13 Li, Y., Shao, M., Lu, S., Chang, C.-C. & Dasgupta, P. K. Variations and sources of ambient formaldehyde for the 2008 Beijing Olympic games. *Atmospheric Environment* **44**, 2632-2639 (2010). https://doi.org/10.1016/j.atmosenv.2010.03.045

14 Pearce, J. L., Beringer, J., Nicholls, N., Hyndman, R. J. & Tapper, N. J. Quantifying the influence of local meteorology on air quality using generalized additive models. *Atmospheric Environment* **45**, 1328-1336 (2011). https://doi.org/10.1016/j.atmosenv.2010.11.051

15 Chaloulakou, A., Kassomenos, P., Spyrellis, N., Demokritou, P. & Koutrakis, P. Measurements of PM_{10} and $PM_{2.5}$ particle concentrations in Athens, Greece. *Atmospheric Environment* **37**, 649-660 (2003). https://doi.org/10.1016/S1352-2310(02)00898-1

16 Schlink, U. *et al.* A rigorous inter-comparison of ground-level ozone predictions. *Atmospheric Environment* **37**, 3237-3253 (2003). https://doi.org/10.1016/S1352-2310(03)00330-3

Part II. Supplementary Tables

Table S1. Variance Inflation Factors (VIFs) of the explanatory variables incorporated in the GAM

Variable	VIF
RH	1.99
BLH	1.92
SSRD	1.90
T	1.75
O3	1.73
EVI	1.63
CC	1.47
CO	1.28
W	1.27
TP	1.23
UVS	1.23
UVD	1.08
DOS	1.02

Table S2. Predictive performance metrics of the GAMs for HCHO concentrations across different site categories

Site type	Concentration type	Correlation coefficient	Deviance explained (%)	RMSE (molec cm^{-2} or ppbv)	MAE (molec cm^{-2} or ppbv)	MAPE (%)
Urban	C_{HCHO}	0.80	64.08	3.77×10^{15}	2.78×10^{15}	30.49
	S_{HCHO}	0.66	45.24	1.85	1.34	61.69
Suburban	C_{HCHO}	0.74	52.29	3.42×10^{15}	2.57×10^{15}	34.70
	S_{HCHO}	0.62	37.69	1.98	1.45	75.61
Background	C_{HCHO}	0.75	56.98	3.43×10^{15}	2.50×10^{15}	32.57
	S_{HCHO}	0.60	35.62	1.69	1.24	66.45

Part III. Supplementary Figures

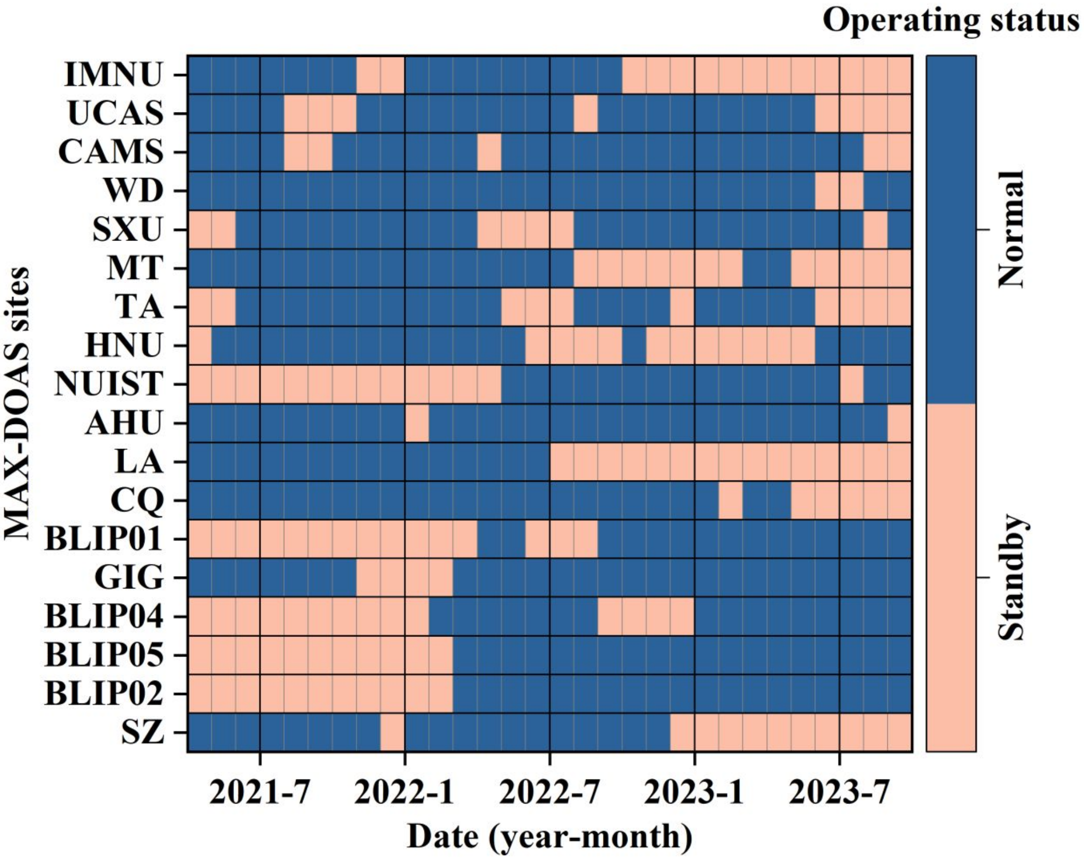

Figure S1. Monitoring status of ground-based MAX-DOAS sites during the study period.

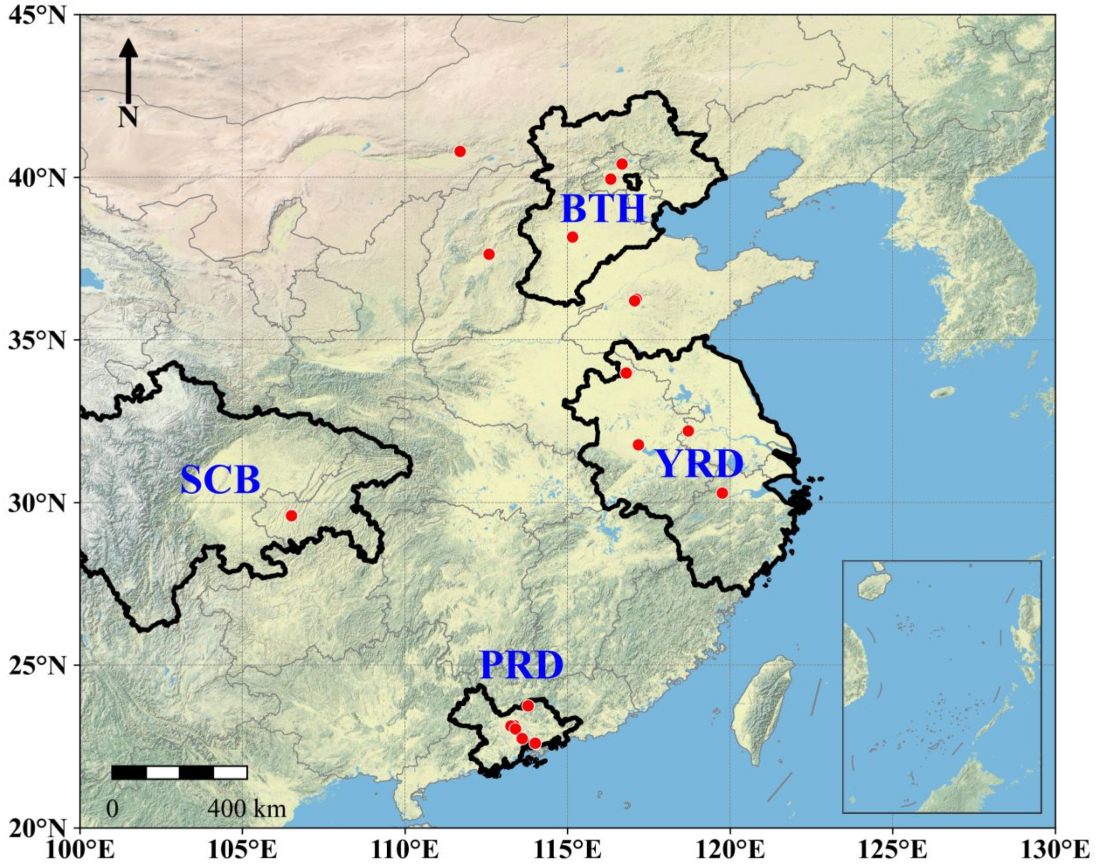

Figure S2. Spatial distribution of ground-based MAX-DOAS sites. Regional boundaries of the Beijing-Tianjin-Hebei (BTH), Yangtze River Delta (YRD), Pearl River Delta (PRD), and Sichuan Basin (SCB) are highlighted.

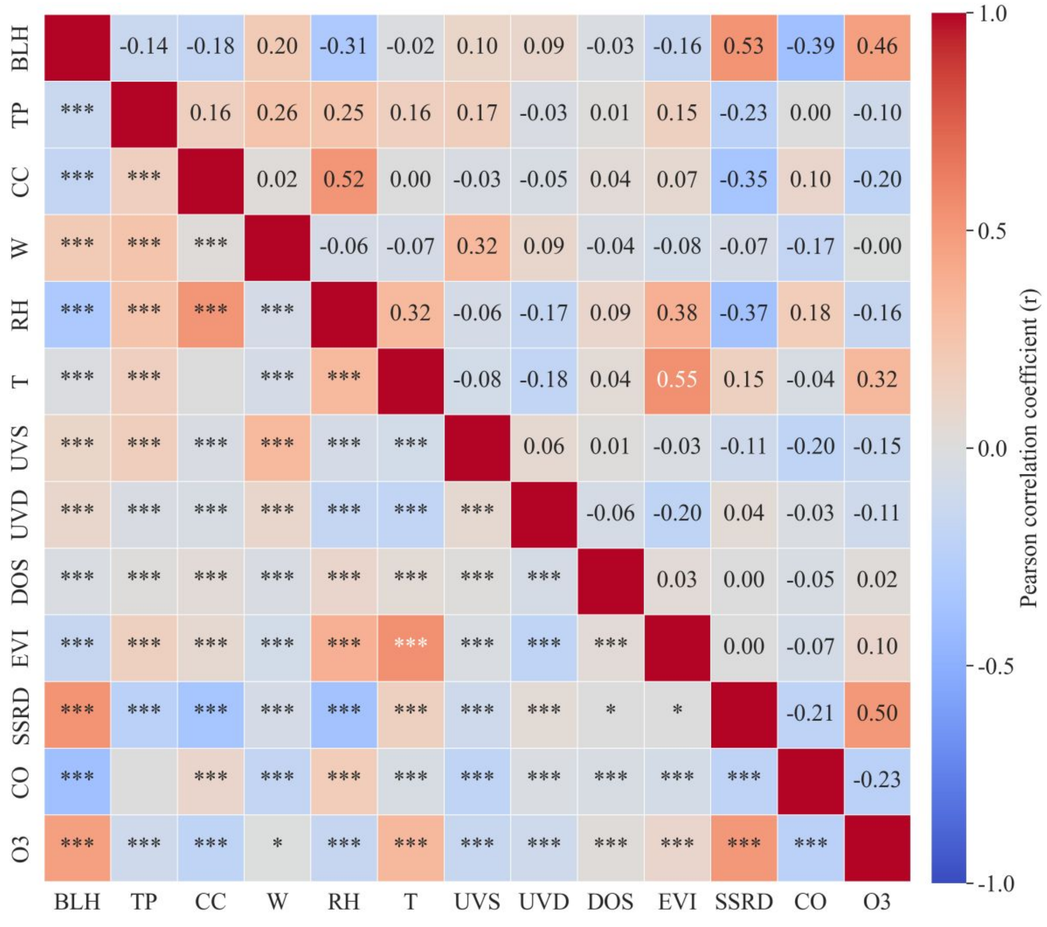

Figure S3. Correlation matrix of explanatory variables in the GAM. The upper triangle shows Pearson correlation coefficients (r), represented by the color scale, while the lower triangle indicates significance levels (*p < 0.05; **p < 0.01; ***p < 0.001).

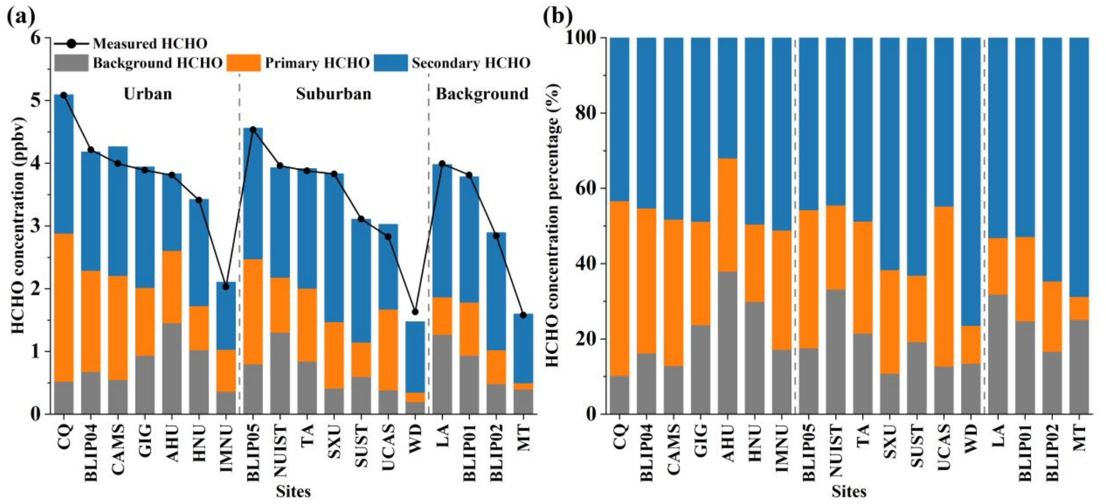

Figure S4. HCHO source apportionment based on Multiple Linear Regression (MLR) at each site. (a) Absolute HCHO composition, where stacked bars represent regression-derived components and the dot-line indicates total HCHO concentrations observed by MAX-DOAS. (b) Relative contributions of each component to total HCHO. Background, primary, and secondary HCHO are

shown in gray, orange, and blue, respectively. Site categories are separated by vertical dashed lines.

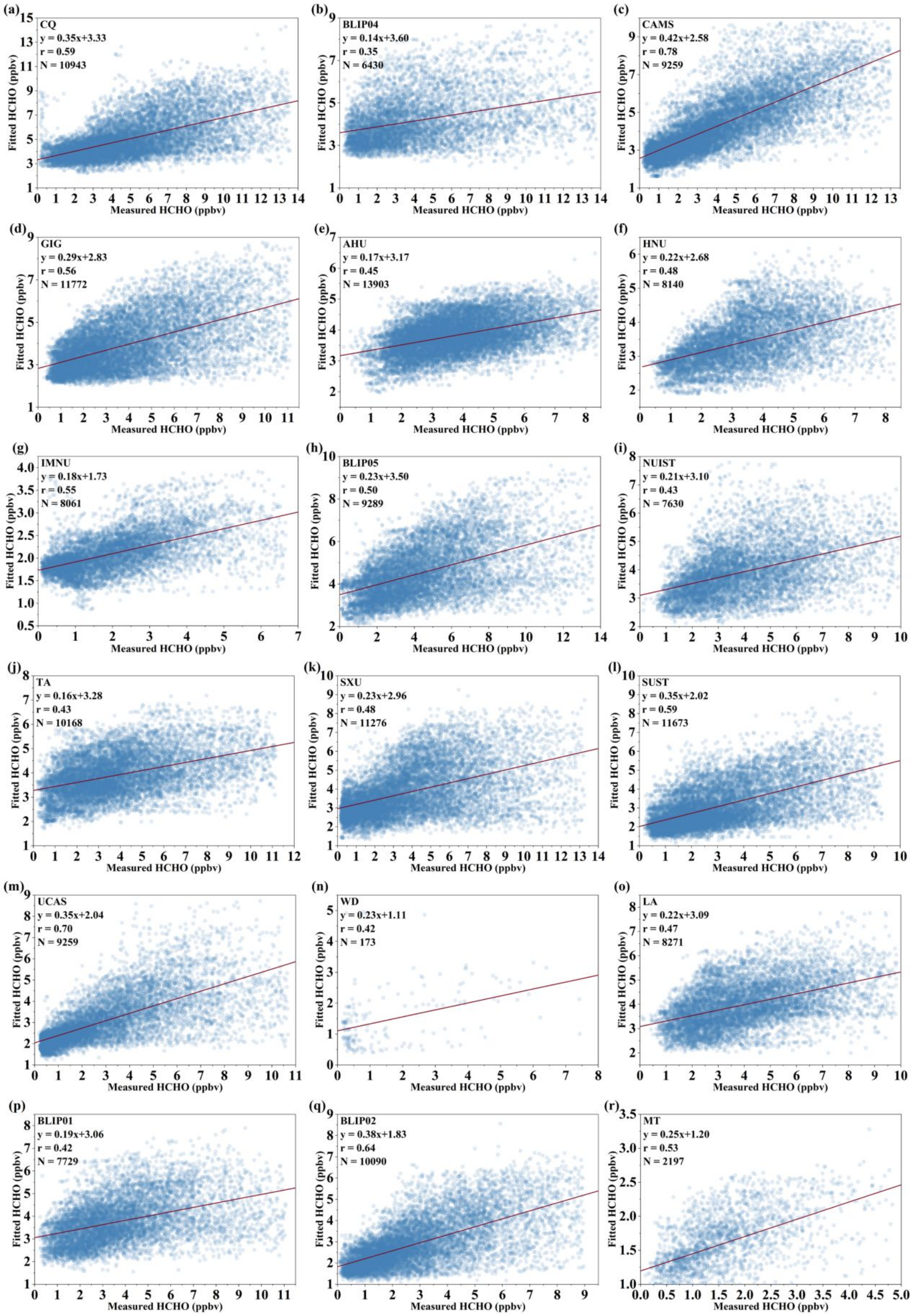

Figure S5. Scatterplots and linear regressions between MAX-DOAS observations and MLR-simulated HCHO concentrations at individual sites. Regression equations, Pearson correlation coefficients (r), and sample sizes (N) are shown in the upper-left corner, while site names are

indicated in the upper-right corner.

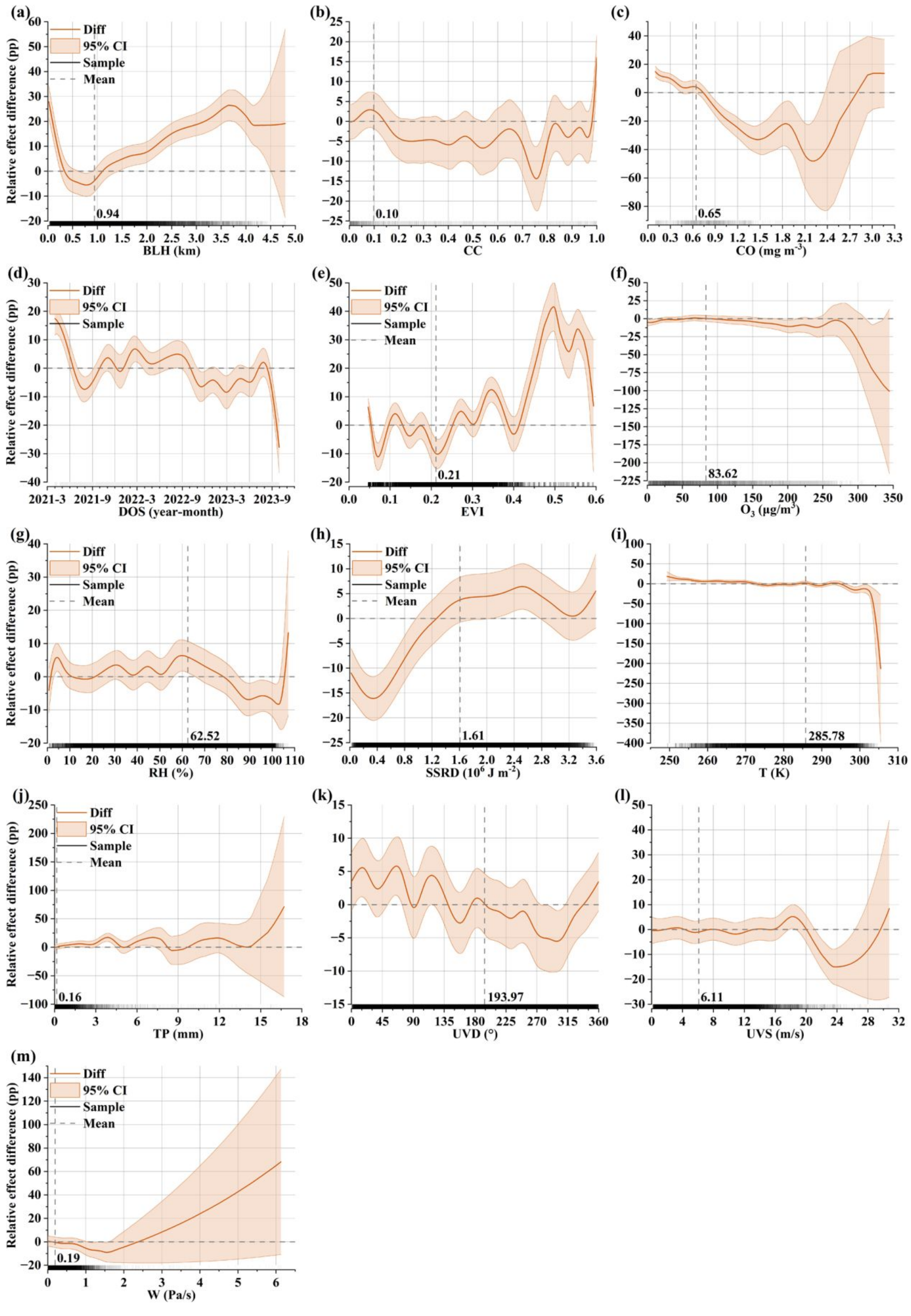

Figure S6. Differences in the relative marginal effects of explanatory variables on C_{HCHO} and S_{HCHO} (C_{HCHO} - S_{HCHO}), defined as Diff (percentage points, pp), at urban sites. Vertical dashed lines indicate the mean values of each variable during the study period.

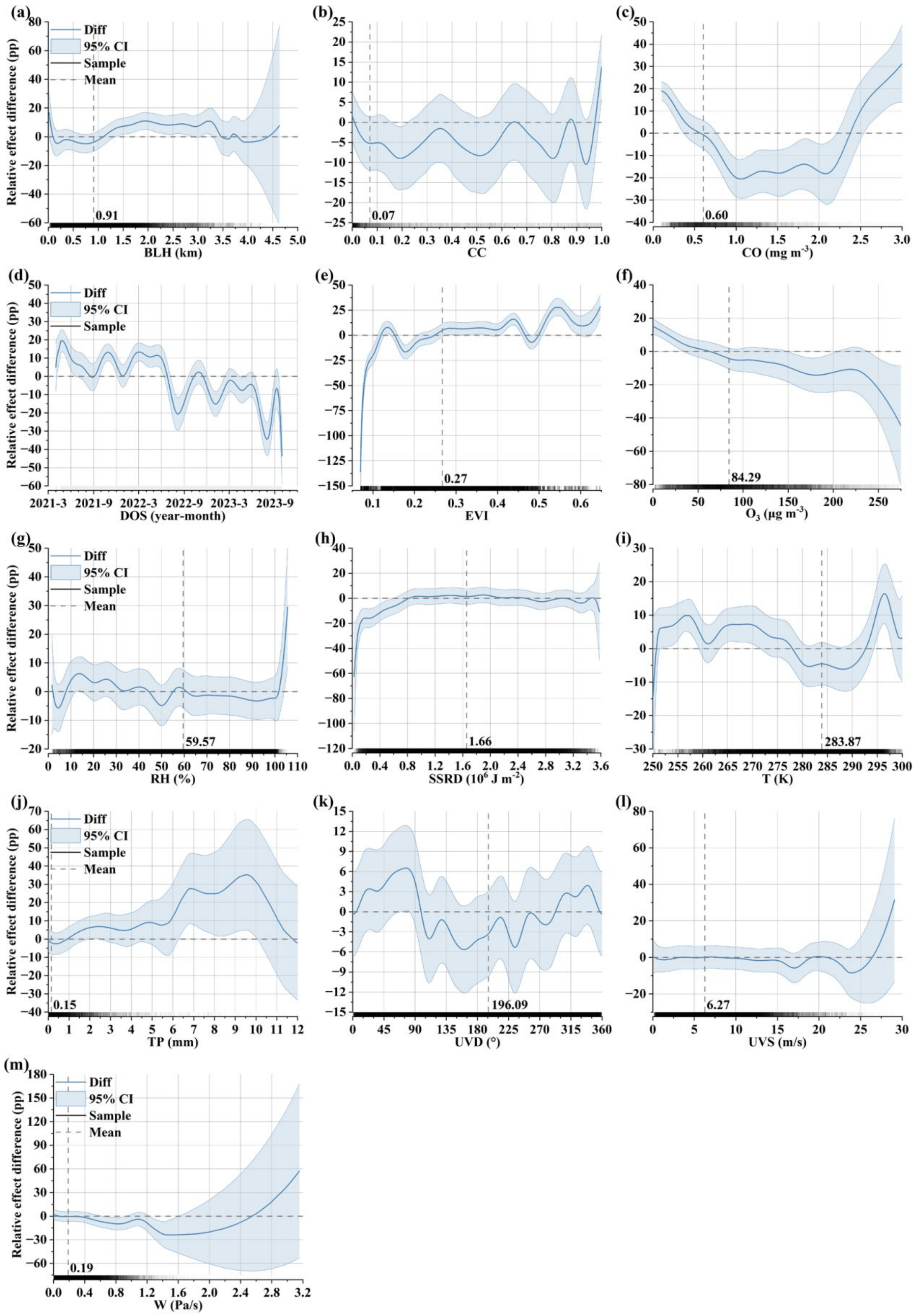

Figure S7. Differences in the relative marginal effects of explanatory variables on C_{HCHO} and S_{HCHO} (C_{HCHO} - S_{HCHO}), defined as Diff (percentage points, pp), at suburban sites. Vertical dashed lines indicate the mean values of each variable during the study period.

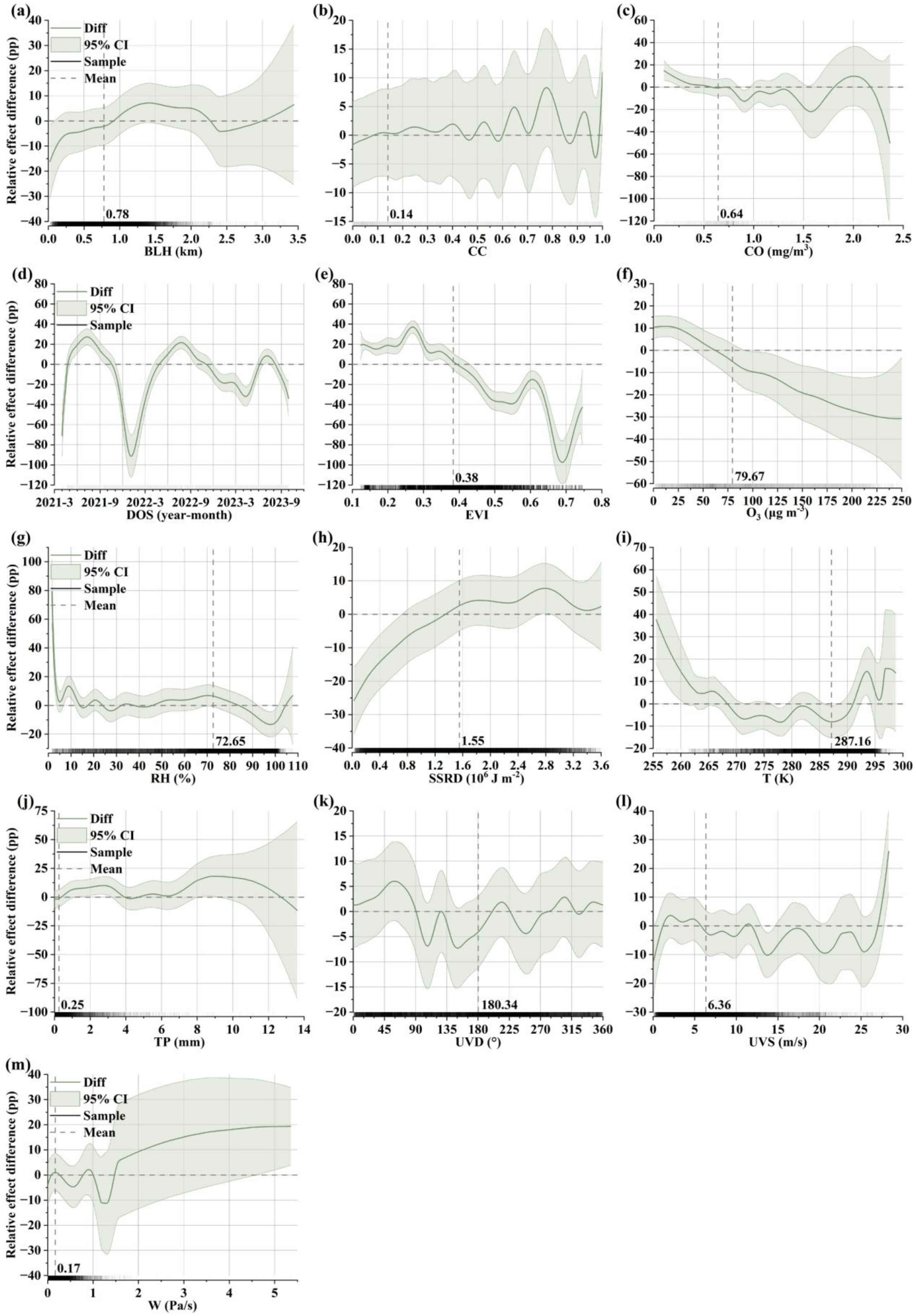

Figure S8. Differences in the relative marginal effects of explanatory variables on C_{HCHO} and S_{HCHO} (C_{HCHO} - S_{HCHO}), defined as Diff (percentage points, pp), at background sites. Vertical dashed lines indicate the mean values of each variable during the study period.

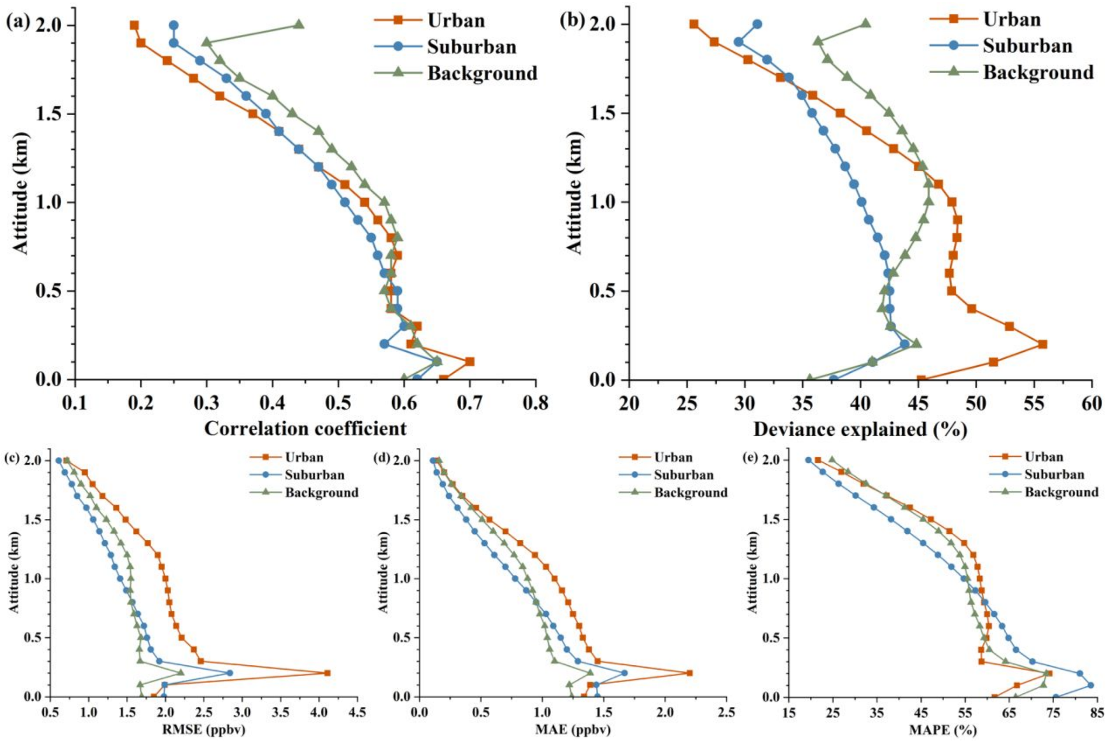

Figure S9. Vertical performance profiles of height-resolved GAMs across site categories. (a) Correlation coefficient (r) between GAM simulations and MAX-DOAS observations; (b) deviance explained (%); (c) Root Mean Square Error (RMSE); (d) Mean Absolute Error (MAE); and (e) Mean Absolute Percentage Error (MAPE). Orange squares, blue circles, and green triangles represent urban, suburban, and background sites, respectively.
